# Serum free thiols predict cardiovascular events and all-cause mortality in the general population: a prospective cohort study

**DOI:** 10.1186/s12916-020-01587-w

**Published:** 2020-05-27

**Authors:** Amaal E. Abdulle, Arno R. Bourgonje, Lyanne M. Kieneker, Anne M. Koning, S. la Bastide-van Gemert, Marian L. C. Bulthuis, Gerard Dijkstra, Klaas Nico Faber, Robin P. F. Dullaart, Stephan J. L. Bakker, Reinold O. B. Gans, Ron T. Gansevoort, Douwe J. Mulder, Andreas Pasch, Harry van Goor

**Affiliations:** 1 Division of Vascular Medicine, Department of Internal Medicine, University of Groningen – University Medical Center Groningen, Groningen, the Netherlands; 2 Department of Gastroenterology and Hepatology, University of Groningen – University Medical Center Groningen, Groningen, the Netherlands; 3 Division of Nephrology, Department of Internal Medicine, University of Groningen – University Medical Center Groningen, Groningen, the Netherlands; 4 Department of Pathology and Medical Biology, Section Pathology, University of Groningen – University Medical Center Groningen, Groningen, the Netherlands; 5 Department of Epidemiology, University of Groningen – University Medical Center Groningen, Groningen, the Netherlands; 6 Division of Endocrinology, Department of Internal Medicine, University of Groningen – University Medical Center Groningen, Groningen, the Netherlands; 7 Institute for Physiology and Pathophysiology, Johannes Kepler University Linz, Linz, Austria

**Keywords:** Oxidative stress, Free thiols, Cardiovascular disease, Mortality, Population study

## Abstract

**Background:**

Serum free thiols (R-SH, sulfhydryl groups) reliably reflect systemic oxidative stress. Since serum free thiols are rapidly oxidized by reactive species, systemic oxidative stress is generally associated with reduced serum free thiol levels. Free thiols associate with favorable disease outcomes in many patient cohorts, and the current hypothesis is that oxidative stress might also play an important role in cardiovascular disease. In this study, we aimed to establish the role of serum free thiols in the general population by investigating their relationship with the risk of cardiovascular (CV) events and all-cause mortality.

**Methods:**

Participants (*n* = 5955) of the Prevention of REnal and Vascular ENd-stage Disease (PREVEND) cohort study from the general population were included. At baseline, serum levels of free thiols were quantified and adjusted to total protein levels. Protein-adjusted serum free thiol levels were studied for their associations with clinical and biochemical parameters, as well as with the risk of CV events and all-cause mortality.

**Results:**

The mean protein-adjusted serum free thiol level was 5.05 ± 1.02 μmol/g of protein. Protein-adjusted serum free thiols significantly predicted the risk of CV events, even after adjustment for potential confounding factors (hazard ratio [HR] per doubling 0.68 [95% confidence interval [CI] 0.47–1.00], *P* = 0.048). Similarly, protein-adjusted serum free thiols were significantly predictive of the risk of all-cause mortality (HR per doubling 0.66 [95% CI 0.44–1.00], *P* = 0.050). Stratified analyses revealed lower HRs for subjects with a lower body mass index (BMI), without hypertension, and without diabetes. Conversely, HRs were lower in subjects with albuminuria.

**Conclusions:**

In this large population-based cohort study, serum free thiols significantly predicted the risk of CV events and all-cause mortality. Our results highlight the potential significance and clinical applicability of serum free thiols since they are amendable to therapeutic intervention.

## Background

Cardiovascular disease (CVD) is among the leading causes of morbidity and mortality globally, and during the past decade, the number of CVD-associated deaths has increased by 12.5% [1, 2]. Although a large proportion of the overall cardiovascular risk can be explained by known risk factors (e.g., smoking, hypertension, obesity, diabetes, and hypercholesterolemia), the pathogenesis of CVD still remains incompletely understood [3]. Current approaches to reduce the CVD burden have focused on encouraging healthier lifestyles. Other approaches have focused on the early identification of patients who are at increased risk of CVD. These latter strategies have faced multiple problems, including limited availability of non-invasive predictive biomarkers to assess the risk of cardiovascular (CV) events or mortality.

Oxidative stress is defined as the imbalance between the production of reactive oxygen species (ROS) and antioxidants. Although ROS, a collective term for oxygen-containing reactive species, are important signaling molecules regulating a variety of physiological processes, overproduction of ROS leads to cellular damage and tissue destruction. Chronic oxidative stress can easily and reliably be monitored as the depletion of serum free thiols, since they are readily oxidized by reactive species [4–6]. In support, overproduction of ROS, as reflected by reduced levels of free thiols, is frequently observed in human disease (like in chronic kidney disease, chronic heart failure, diabetes, cancer, systemic sclerosis, and Crohn’s disease) that is typically associated with oxidative stress [7–10]. Free thiol groups (R-SH, sulfhydryl groups) are thought to play a protective role against oxidative stress through ROS scavenging and are an important component of the in vivo antioxidant buffer capacity. Studies from our center have demonstrated that higher levels of serum free thiols (i.e., lower levels of oxidative stress) are associated with favorable outcomes in patients with chronic heart failure [7], as well as with a beneficial outcome and cardiovascular risk profile in renal transplant recipients [11].

Oxidative stress has been acknowledged to play an important role in CVD, and conditions promoting oxidative stress have also been associated with risk of CVD [12]. Therefore, we hypothesized that serum free thiol groups, as overall markers of systemic oxidative stress, might have merit as a read-out of cardiovascular health in the general population. Moreover, free thiols might be a valuable indicator of the risk of all-cause mortality and risk of CV events, which could be applicable for risk stratification aimed to reduce CVD burden. Therefore, the aim of this study was to determine the predictive value of serum levels of free thiols regarding the risk of cardiovascular events and all-cause mortality in the general population.

## Methods

### Study population

This study used data from the Prevention of REnal and Vascular ENd-stage Disease (PREVEND) cohort study [13]. This is a large, prospective population-based cohort study with participants from the northern part of the Netherlands. The PREVEND study was set up to investigate cardiovascular and renal disease outcomes focused on albuminuria as the main risk factor. From 1997 to 1998, 85,421 inhabitants aged 28–75 years from the northern parts of the Netherlands received a questionnaire asking information about demographics, medication use, cardiovascular disease, and pregnancy, including a request to supply an early morning urine sample. Participants who had a previous diagnosis of type 1 diabetes mellitus and insulin-treated type 2 diabetes mellitus and pregnant women were excluded from the study. In total, 40,856 subjects responded to the questionnaire and were analyzed for urinary albumin concentrations. Study participants having urinary albumin concentrations ≥ 10 mg/L (*n* = 6000) were invited to visit the outpatient research clinic of the University Medical Center Groningen (UMCG), as well as a random selection of participants with urinary albumin concentrations < 10 mg/L (*n* = 2592). Therefore, the PREVEND study consisted of an initial total of 8592 participants who completed the full study program [14]. However, serum samples from 6136 participants were collected during the second visit (2001–2003, referred to as the baseline visit in the present study). Participants who had a CV event between visit 1 and visit 2 (*n* = 181) were excluded from the analyses given the fact that re-events were not registered for these subjects. However, participants with a known cardiovascular disease prior to visit 2, who did not experience a CV event between visit 1 and visit 2, were included. Therefore, a total of 5955 participants were included in the current study. This study was approved by the Institutional Review Board (IRB) (full name in Dutch: “Medisch Ethische Toetsingscommissie” [METc]) of the UMCG. The study was conducted in accordance with the principles of the Declaration of Helsinki (2013), and all study participants provided written informed consent.

### Data collection

All study participants visited the outpatient research clinic of the University Medical Center Groningen (UMCG), Groningen, the Netherlands. During the first visit, participants were requested to complete a questionnaire containing information about demographics, health status, history of cardiovascular disease (CVD), medication usage, and lifestyle habits (e.g., smoking habits), and anthropometric measurements were performed as well. At the second visit, systolic and diastolic blood pressure was automatically measured every minute for a period of 8 min in a supine position (Dinamap XL Model 9300 series device, Johnson & Johnson Medical, Tampa, FL). Blood pressure was defined as the average of the last two measurements. Furthermore, serum samples were collected, and patients were asked to collect 24-h urine after they were provided with both oral and written instructions. Serum samples were stored at − 80 °C, while urine samples were stored at − 20 °C until further analysis.

### Laboratory measurements

High-sensitive C-reactive protein (hsCRP) and urinary albumin excretion (UAE) were measured by nephelometry (Dade Behring Diagnostics, Marburg, Germany). UAE was measured twice in two different 24-h urine collections, and the average was taken for analyses. Serum total cholesterol and fasting glucose were measured by dry chemistry (Eastman Kodak, Rochester, NY, USA). Serum total protein was measured using spectrophotometry (Roche Modular, Roche, Mannheim, Germany). High-density lipoprotein (HDL) cholesterol was measured using a homogeneous method (direct HDL, AerosetTM System, Abbott Laboratories, Abbott Park, IL, USA). Low-density lipoprotein (LDL) cholesterol was determined by the Friedewald formula (if triglycerides ≤ 4.5 mmol/L). Triglycerides were measured with an enzymatic method. Serum creatinine was measured enzymatically (Roche Modular, Roche Diagnostics, Mannheim, Germany). Serum cystatin C was measured using the Gentian Cystatin C Immunoassay (Gentian AS, Moss, Norway) on a modular analyzer (Roche Diagnostics). Cystatin C was directly calibrated using standards from the manufacturer’s supplies (according to the International Federation of Clinical Chemistry Working Group for Standardization of Serum Cystatin C) [15].

### Serum free thiol measurements

Measurement of serum free thiols (R-SH, sulfhydryl groups) was performed as previously described, with minor modifications [16, 17]. After thawing, samples were 4-fold diluted using 0.1 M Tris buffer (pH 8.2). Background absorption was measured at 412 nm using the Varioskan microplate reader (Thermo Scientific, Breda, the Netherlands), together with a reference measurement at 630 nm. Subsequently, 20 μL 1.9 mM 5,5′-dithio-bis (2-nitrobenzoic acid) (DTNB, Ellman’s reagent, CAS no. 69-78-3, Sigma Aldrich Corporation, St. Louis, MO, USA) was added to the samples in 0.1 M phosphate buffer (pH 7.0). After an incubation time of 20 min at room temperature, absorbance was measured again. The final serum free thiol concentrations were established by parallel measurement of an l-cysteine (CAS no. 52-90-4, Fluka Biochemika, Buchs, Switzerland) calibration curve (range from 15.6 to 1000 μM) in 0.1 M Tris/10 mM EDTA (pH 8.2). Intra- and interassay coefficients of variation (CV) of serum free thiol measurements were 1.9% and 5.0%, respectively. Finally, serum free thiol concentrations were adjusted to total serum protein (measured according to standard procedures) by calculating the free thiol/total protein ratio (μmol/g of protein). This adjustment was performed since serum proteins harbor the largest amount of free thiols and therefore greatly determine the quantity of potentially detectable free thiol groups [18].

### Study outcomes and definitions

Estimated glomerular filtration rates (eGFR) were calculated using the combined creatinine cystatin C-based Chronic Kidney Disease Epidemiology Collaboration (CKD-EPI) equation [19]. Type 2 diabetes mellitus (T2DM) was defined as a fasting glucose level ≥ 7.0 mM or the use of antidiabetic medications according to the guidelines of the American Diabetic Association (ADA). Hypercholesterolemia was defined as a serum total cholesterol level > 6.5 mmol/L, serum HDL cholesterol level < 0.9 mmol/L, or the use of lipid-lowering drugs at baseline. Fatal and non-fatal cardiovascular events (CV) (i.e., combined outcomes of acute myocardial infarction (AMI), acute or subacute ischemic heart disease (IHD), coronary artery bypass grafting (CABG), percutaneous transluminal coronary angioplasty (PTCA), intracerebral hemorrhage, other intracranial hemorrhages, subarachnoid hemorrhage, occlusion and stenosis of precerebral or cerebral arteries, and other vascular interventions such as carotid desobstruction, aorta peripheral bypass surgery, or percutaneous transluminal femoral angioplasty) and all-cause mortality were considered primary endpoints of the current study. These outcomes were determined by using information from the Dutch National Registry of all hospital discharge diagnoses (Prismant). This information was classified in accordance with the International Statistical Classification of Diseases (ICD-10) and the International Classification of Health Interventions [20]. In addition, the classical 3-point major adverse cardiovascular events (3P-MACE) was considered as the composite endpoint for CV events, consisting of fatal CV events, non-fatal myocardial infarction, and non-fatal stroke [21].

### Statistical analysis

Data analysis was performed using the SPSS Statistics 25.0 software package (SPSS Inc., Chicago, IL, USA), Stata 15.1 (Stata Corp LLC, College Station, TX, USA), and R version 3.5.2. (Vienna, Austria). Data visualization was performed using GraphPad Prism 5.0 (GraphPad Software, San Diego, CA, USA) and RStudio (version 1.2.1335; RStudio, Boston, MA). Demographic and clinical characteristics were presented as mean ± standard deviation (SD), median (interquartile range [IQR]) in case of skewed variables, or proportions *n* with corresponding percentages (%). Normality was assessed and visualized using histograms and normal probability plots (Q-Q plots). Between-group comparisons for continuous variables were performed using one-way analysis of variance (ANOVA) or Kruskal-Wallis tests, while for nominal variables, chi-square tests were performed, as appropriate. Protein-adjusted serum free thiol concentrations were ^2^log-transformed to facilitate result interpretation (per doubling). Survival distributions were assessed for tertiles of protein-adjusted serum free thiol concentrations using Kaplan-Meier curves and compared with log-rank tests. Survival time was defined from baseline (time of serum sample collection) until the date of the last examination that participants attended: the incidence of the cardiovascular (CV) event, death, or January 1, 2010 (end of follow-up). Cox proportional hazards regression analyses were performed to assess the association between protein-adjusted serum free thiols and the risk of CV events or all-cause mortality. The results were expressed as hazard ratios (HRs) with corresponding 95% confidence intervals. Univariable associations were followed by multivariable Cox regression models to adjust for potential confounding factors and stratified analyses to assess HRs across several subgroups. To evaluate the discriminative capacity of Cox regression models, receiver operating characteristics (ROC) analyses were performed by calculating the Harrell C index for the various models. The Harrell C index represents the area under the ROC curve (AUC) and is compatible with time-to-event data. Finally, Cox regression analyses were repeated with restricted cubic splines with three knots to evaluate potential non-linearity of associations between protein-adjusted serum-free thiols and CV events or all-cause mortality. Non-linearity was evaluated by likelihood ratio tests, where nested models were compared using linear or linear and cubic spline terms. In addition, a sensitivity analysis was performed in which oversampling of subjects with higher urinary albumin concentrations was addressed by using design-based Cox proportional hazards regression models. Two-tailed *P* values ≤ 0.05 were considered statistically significant.

### Selection of confounding variables

In line with the guidelines for adjusting for confounders as described previously, we used causal models (directed acyclic graphs (DAGs) and their associated theory) to distinguish the appropriate set of confounders for estimating our effect of interest [22–24]. Based on previously established theory in our field and respecting constraints imposed by time and logic, the DAG represents the causal mechanisms we hypothesize to be underlying the variables at hand (scenario 1) (Additional file 1: Figure S1) [9, 25–31].

Arrows depict hypothesized causal (direct) effects between variables, whereas the absence of an arrow between two variables represents the assumption of no such direct effect. Focusing on the effect of oxidative stress on the outcome (cardiovascular events and all-cause mortality), we can now identify those variables for which conditioning in the analysis is necessary in order to obtain an unconfounded effect estimate in our statistical analysis. This is performed by determining unblocked causal paths in the graph from oxidative stress to outcome, which—when left unblocked—would bias the desired effect estimate. Consequently, we concluded that conditioning on the variables gender, age, smoking, systolic blood pressure, total cholesterol, diabetes, and hsCRP (as a proxy for inflammation) in the analysis would provide an unconfounded effect estimate of oxidative stress on the outcome.

As there could also be arguments that would lead the arrow pointing from hsCRP (as a proxy for inflammation) to oxidative stress to point the other way around [32], we additionally included BMI in our set of confounders to eliminate additional potential bias introduced by that scenario (scenario 2). It has to be noted that including BMI in the DAG (scenario 1) should causally have no influence on the effect estimation (Additional file 1: Figure S1).

## Results

### Patient characteristics

**Fig. 1:**
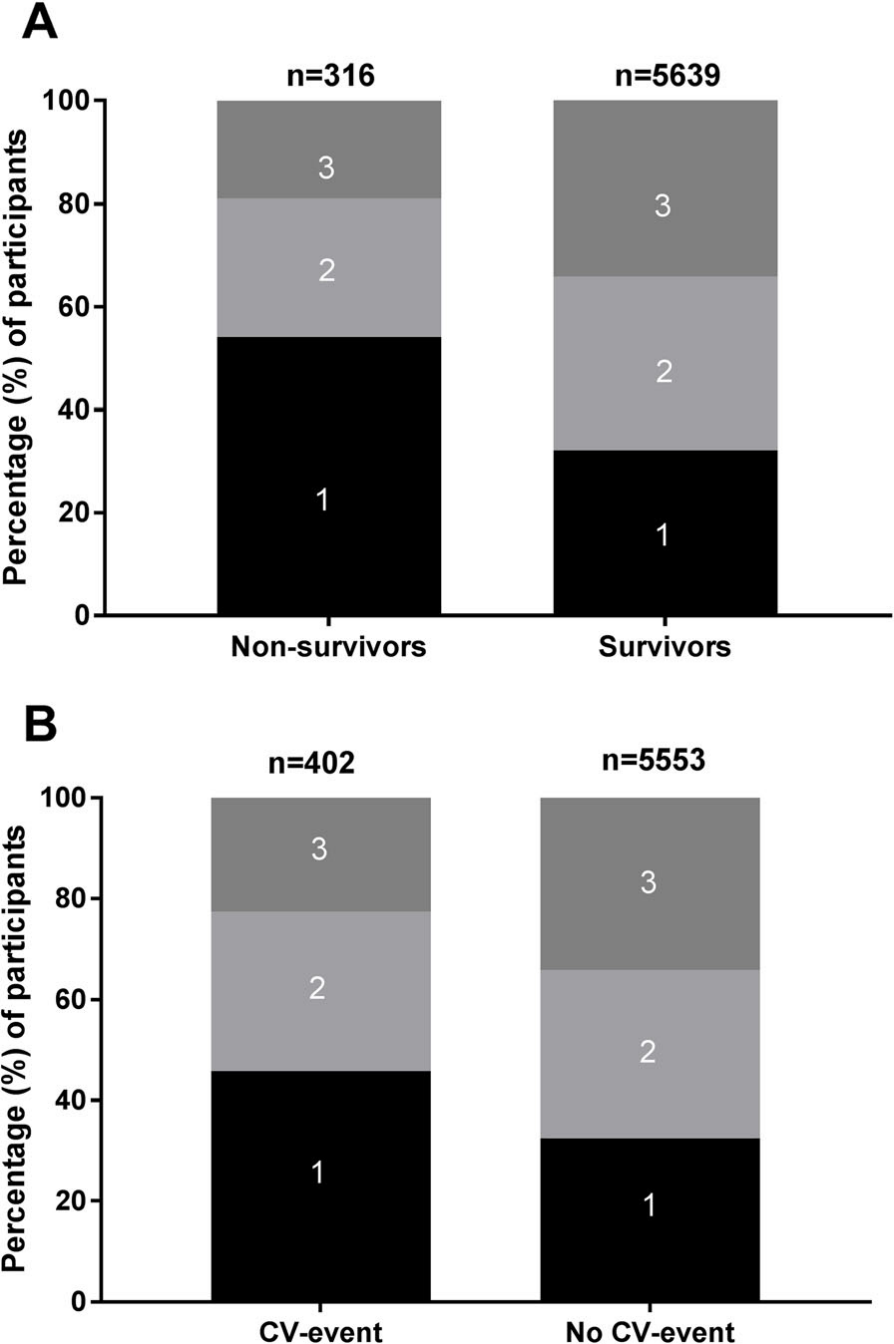
Distributions of tertiles of protein-adjusted serum free thiol concentrations among survivor and non-survivors (**a**) and subjects with and without a CV event (**b**). Tertile 1, protein-adjusted serum free thiol concentration range < 4.65; tertile 2, protein-adjusted serum free thiol concentration range 4.65–5.45; tertile 3, protein-adjusted serum free thiol concentration range > 5.46

The baseline characteristics of the study population are presented in Table 1. The mean age was 51.6 (43.3–61.7) years, and 51% of the participants were female. Participants in the lowest tertile of protein-adjusted serum free thiols were significantly older and had a higher body mass index (BMI) as compared to the other groups (both *P* < 0.001). A history of cardiovascular disease was observed in 3.6% of the total cohort, and diabetes was found in 2.4% of the total number of participants. Mean protein-adjusted serum free thiol level was 5.05 ± 1.02 μmol/g of protein (median 5.07 μmol/g of protein, full range 1.47–9.33 μmol/g). Cardiovascular diseases and diabetes were more frequently observed in subjects within the lowest free thiol tertile (both *P* < 0.001). Higher rates of mortality (*P* < 0.001) and cardiovascular events (*P* < 0.001) were more often observed in subjects within the lowest tertile of serum free thiols. Figure 1 shows the distribution of serum free thiol tertiles among survivors and non-survivors and among subjects with and without a CV event.

**Table 1:** Demographic and clinical characteristics of the study population

	Total, *N* = 5955	T1, *N* = 1985	T2, *N* = 1985	T3, *N* = 1985	*P* value
Serum free thiol (μmol/g) levels (adjusted for protein)	5.05 (± 1.02) μmol/g	< 4.65 μmol/g	4.65–5.46 μmol/g	> 5.46 μmol/g	
Age (years)	51.6 (43.3–61.7)	57.6 (47.8–66.6)	51.1 (43.4–59.8)	47.7 (41.1–55.3)	**< 0.001**
Female, *n* (%)	3038 (51.0%)	1084 (54.6%)	1013 (51.0%)	941 (47.4%)	**< 0.001**
Blood pressure					
SBP (mmHg)	126 (± 19)	129 (± 20)	125 (± 18)	123 (± 17)	**< 0.001**
DBP (mmHg)	73 (± 9)	74 (± 9)	73 (± 9)	73 (± 9)	**< 0.001**
Heart rate (bpm)	68 (± 10)	69 (± 11)	69 (± 10)	68 (± 10)	0.44
Obesity variables					
BMI (kg/m^2^)	26.0 (23.6–28.9)	26.8 (24.3–29.9)	26.0 (23.6–28.7)	25.3 (23.0–28.0)	**< 0.001**
Waist circumference (cm)	91.0 (82.0–100.0)	94.0 (85.0–102.0)	91.0 (82.0–100.0)	89.0 (81.0–98.0)	**< 0.001**
Smoking, *n* (%)					**< 0.001**
Never, *n* (%)	1757 (29.5%)	583 (29.4%)	578 (29.1%)	596 (30.0%)	
Current, *n* (%)	1655 (27.8%)	494 (24.9%)	538 (27.1%)	623 (31.4%)	
Former, *n* (%)	2472 (41.5%)	887 (44.7%)	848 (42.7%)	737 (37.1%)	
History of cardiovascular disease, *n* (%)	217 (3.6%)	100 (5.0%)	69 (3.5%)	48 (2.4%)	**< 0.001**
Diabetes, *n* (%)	144 (2.4%)	76 (3.8%)	34 (1.7%)	34 (1.7%)	**< 0.001**
Medication, *n* (%)					
Antihypertensive drugs, *n* (%)	1037 (17.4%)	481 (24.2%)	307 (15.5%)	249 (12.5%)	**< 0.001**
Lipid-lowering drugs, *n* (%)	371 (6.2%)	155 (7.8%)	126 (6.3%)	90 (4.5%)	**< 0.001**
Antidiabetic drugs, *n* (%)	78 (1.3%)	40 (2.0%)	19 (1.0%)	19 (1.0%)	**0.006**
Laboratory measurements					
Total cholesterol (mmol/L)	5.5 (± 1.0)	5.5 (± 1.0)	5.5 (± 1.0)	5.4 (± 1.1)	**< 0.001**
HDL (mmol/L)	1.2 (1.0–1.5)	1.2 (1.1–1.5)	1.2 (1.0–1.4)	1.2 (1.0–1.4)	0.68
LDL (mmol/L)	3.4 (2.7–4.2)	3.5 (2.8–4.2)	3.3 (2.7–4.1)	3.3 (2.6–4.1)	0.20
Triglycerides (mmol/L)	1.1 (0.8–1.6)	1.1 (0.8–1.6)	1.1 (0.8–1.6)	1.0 (0.8–1.6)	**0.001**
hsCRP (mg/L)	1.3 (0.6–2.9)	1.6 (0.8–3.9)	1.3 (0.6–2.8)	1.0 (0.5–2.3)	**< 0.001**
Fasting glucose (mmol/L)	4.7 (4.4–5.2)	4.8 (4.5–5.3)	4.7 (4.4–5.2)	4.7 (4.4–5.2)	**< 0.001**
eGFR (mL/min/1.73 m^2^)	94.5 (82.2–104.7)	86.5 (74.7–99.0)	95.0 (83.9–14.0)	99.8 (90.1–108.2)	**< 0.001**
Albuminuria (mg/L)	8.5 (6.0–15.0)	9.3 (6.2–19.0)	8.4 (6.0–14.0)	8.1 (5.95–13.1)	**< 0.001**
CV events, *n* (%)	402 (6.8%)	184 (9.3%)	127 (6.4%)	91 (4.6%)	**< 0.001**
Mortality, *n* (%)	316 (5.3%)	171 (8.6%)	85 (4.3%)	60 (3.0%)	**< 0.001**

Data are presented as mean ± SD, proportions *n* with corresponding percentages (%) or median (IQR)

*P* values in **bold** indicate statistical significance

*SBP* systolic blood pressure, *DBP* diastolic blood pressure, *BMI* body mass index, *HDL* high-density lipoprotein, *LDL* low-density lipoprotein, *hsCRP* high-sensitive C-reactive protein, *eGFR* estimated glomerular filtration rate, *CV* cardiovascular

### Protein-adjusted serum free thiols and risk of cardiovascular events and all-cause mortality

**Fig. 2:**
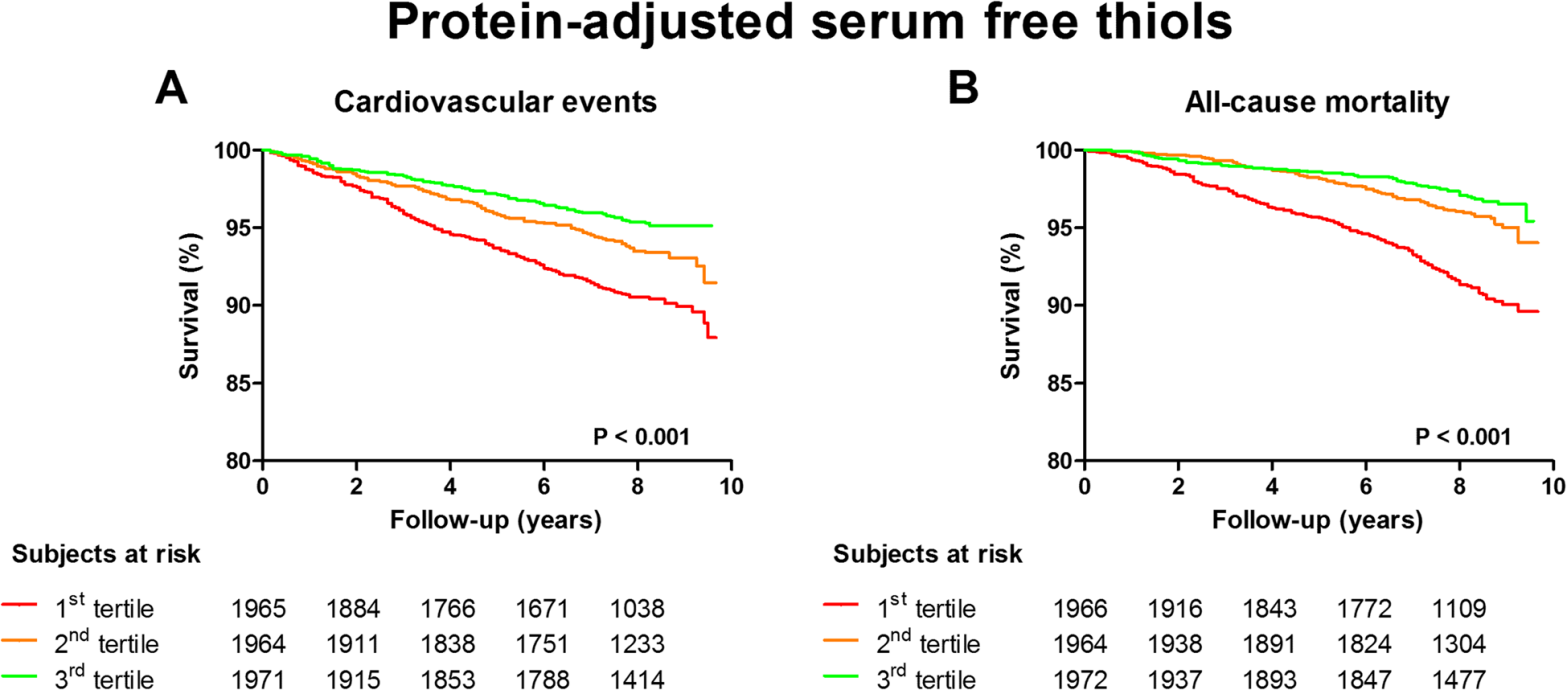
Kaplan-Meier survival distributions for tertiles of protein-adjusted serum free thiol concentrations. **a** Kaplan-Meier curve representing cardiovascular disease-free survival with the highest rate of cardiovascular events occurring in the lowest tertile of protein-adjusted serum free thiols (log-rank test, *P* < 0.0001). **b** Kaplan-Meier curve representing survival with the highest mortality rate occurring in the lowest tertile of protein-adjusted serum free thiols (log-rank test, *P* < 0.0001)

**Fig. 3:**
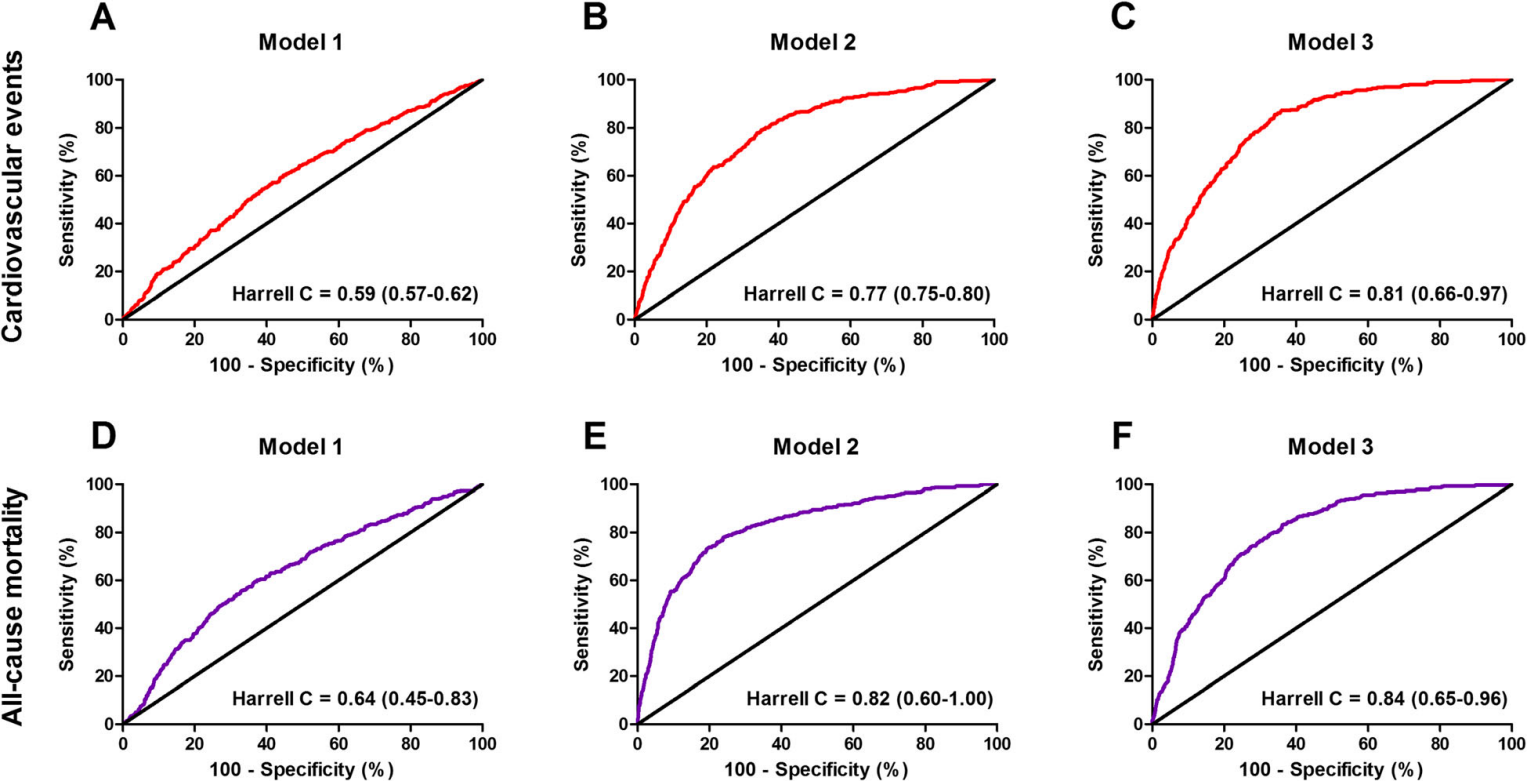
**a**–**f** Receiver operating characteristic (ROC) curves with associated Harrell C indices (95% confidence interval [CI]) of the different Cox proportional hazards regression models. **a**–**c** Discriminative ability of Cox regression models for the association between protein-adjusted serum free thiols and cardiovascular events. **d**–**f** Discriminative ability of Cox regression models for the association between protein-adjusted serum free thiols and all-cause mortality

The mean follow-up of study participants was 7.7 ± 2.0 years, during which 402 (6.8%) cardiovascular events occurred. The highest frequency of cardiovascular events was observed in participants within the lowest tertile of protein-adjusted serum free thiols (184 [9.3%], *P* < 0.001). Kaplan-Meier survival analysis showed a significant difference in the survival distribution between tertiles of protein-adjusted serum free thiols among participants with and without cardiovascular events (Fig. 2a, *P* < 0.0001, log-rank test). Cox proportional hazards regression analyses and corresponding Harrell C indices demonstrated a statistically significant prediction of the risk of cardiovascular events (Table 2 (A), model 1, hazard ratio [HR] per doubling of concentration 0.39 [0.29–0.51], *P* < 0.001). After adjustment for potential confounding factors (age, sex, systolic blood pressure, BMI, total cholesterol, diabetes, current smoking, and hsCRP levels), this predictive association remained statistically significant (Table 2 (A), model 3, HR per doubling of concentration 0.68 [0.47–1.00], *P* = 0.048). These results remained unchanged when the 3P-MACE was considered as the composite endpoint. Discriminative capacity of Cox regression models significantly improved after adjustment for potential confounding factors (Fig. 3a–c).

**Table 2:** Cox proportional hazards regression models of the association between protein-adjusted serum-free thiols and potential confounding factors with (A) cardiovascular events and (B) all-cause mortality

	HR per doubling	Tertiles of protein-adjusted serum-free thiols		
		< 4.65 μmol/g	4.65–5.46 μmol/g	> 5.46 μmol/g
A. Cardiovascular events				
Model 1	0.39 (0.29–0.51), ***P*** **< 0.001**	1.00 (reference)	0.66 (0.53–0.83), ***P*** **< 0.001**	0.47 (0.37–0.61), ***P*** **< 0.001**
Model 2	0.71 (0.51–0.97), ***P*** **= 0.031**	1.00 (reference)	0.94 (0.75–1.19), *P* = 0.600	0.82 (0.63–1.07), *P* = 0.135
Model 3	0.68 (0.47–1.00), ***P*** **= 0.048**	1.00 (reference)	1.00 (0.77–1.30), *P* = 0.998	0.81 (0.59–1.10), *P* = 0.178
B. All-cause mortality				
Model 1	0.30 (0.22–0.41), ***P*** **< 0.001**	1.00 (reference)	0.47 (0.37–0.61), ***P*** **< 0.001**	0.34 (0.25–0.45), ***P*** **< 0.001**
Model 2	0.71 (0.50–1.01), *P* = 0.056	1.00 (reference)	0.77 (0.59–1.00), ***P*** **= 0.048**	0.73 (0.54–1.00), ***P*** **= 0.049**
Model 3	0.66 (0.44–1.00), ***P*** **= 0.050**	1.00 (reference)	0.87 (0.65–1.16), *P* = 0.337	0.68 (0.47–0.98), ***P*** **= 0.037**

Model 1: crude; model 2: model 1, age- and sex-adjusted; model 3 (based on DAG): model 2, hsCRP, current smoking, systolic blood pressure, total cholesterol, diabetes, and BMI

*P* values in **bold** indicate statistical significance

*HR* hazard ratio

During follow-up, 316 (5.3%) subjects died. The highest mortality rate occurred in the lowest tertile of protein-adjusted serum-free thiols (171 [8.6%], *P* < 0.001). Kaplan-Meier survival analysis showed a significantly differential survival distribution between tertiles of protein-adjusted serum free thiols among survivors and non-survivors (Fig. 2b, *P* < 0.0001, log-rank test). Cox proportional hazards regression analyses and corresponding Harrell C indices showed a significant inverse predictive association between protein-adjusted serum free thiol concentrations and the risk of all-cause mortality (Table 2 (B), model 1, HR per doubling of concentration 0.30 [0.22–0.41], *P* < 0.001). This association remained significant after adjustment for potential confounders (Table 2 (B), model 3, 0.66 [0.44–1.00], *P* = 0.050). Discriminative capacity of Cox regression models significantly improved after adjustment for potential confounding factors (Fig. 3d–f). Restricted cubic splines showed no significant deviances from linear associations with either the incidence of CV events or all-cause mortality for protein-adjusted serum free thiol concentrations (Additional file 2: Figure S2A-B, *P* = 0.258 and *P* = 0.642, respectively). In weighted analyses, in which we accounted for the oversampling of subjects with higher urinary albumin concentrations, the results (free thiols per doubling) differed slightly (CV events, 0.65 [95% CI 0.38–1.10]; all-cause mortality, 0.59 [95% CI 0.34–1.02]).

### Stratified analyses

**Fig. 4:**
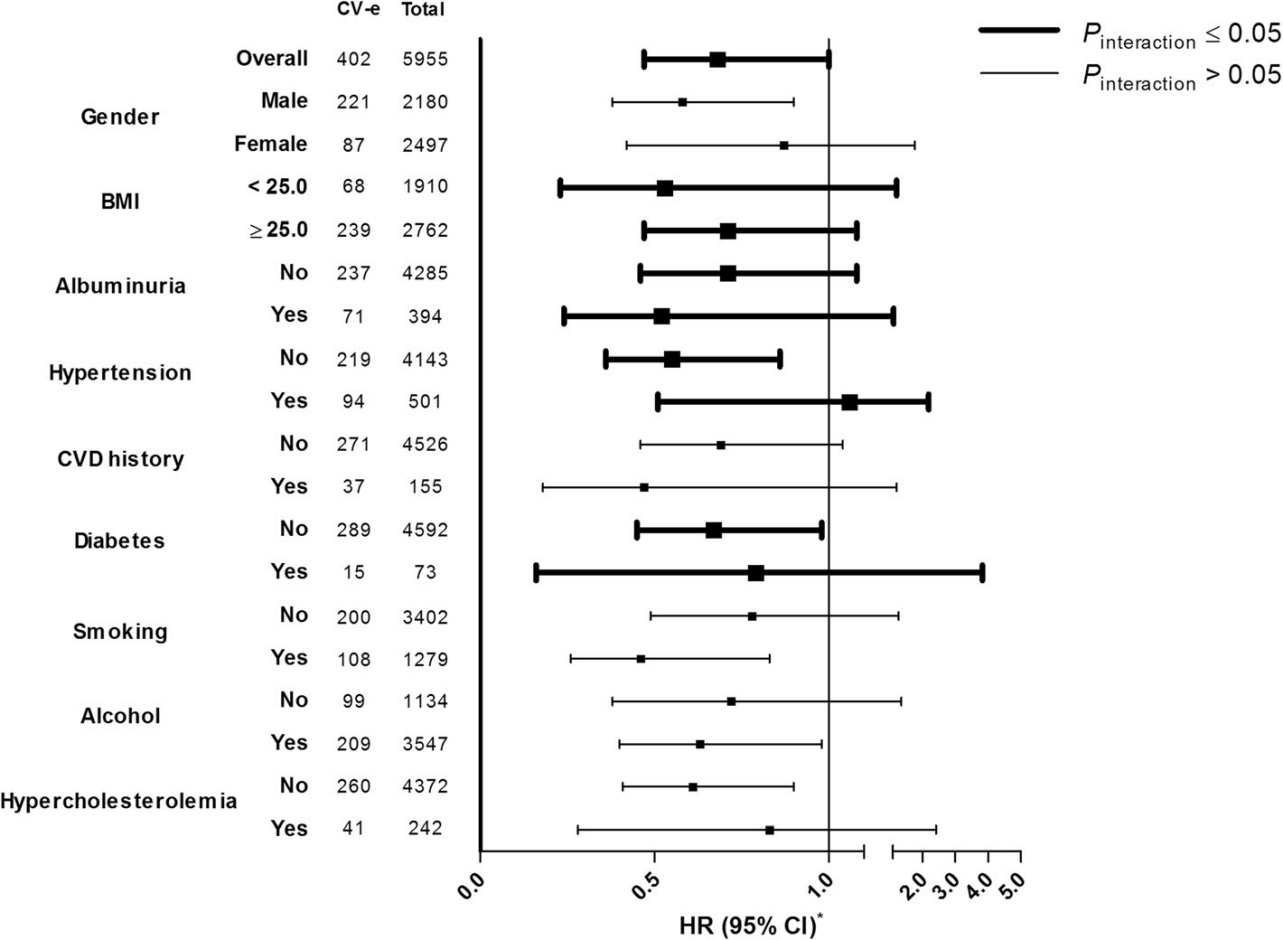
Associations between protein-adjusted serum free thiol levels and the risk of cardiovascular (CV) events across various subgroups. Hazard ratios (HRs) are shown with corresponding 95% confidence intervals (CI). HRs represent consistently inverse associations across subgroups, with the exception of stratification by the presence of hypertension (*P*_interaction_ < 0.001). Stratifications by BMI, albuminuria, hypertension, diabetes, and hypercholesterolemia show significant interactions. Corresponding HRs are consistently lower for subjects with a more favorable cardiovascular risk profile (i.e., lower BMI and the absence of hypertension and diabetes), whereas the presence of CVD history or albuminuria shows lower HRs as well. *HRs adjusted for potential confounding factors (sex, age, hsCRP, current smoking, systolic blood pressure, total cholesterol, diabetes, and BMI). CV-e, cardiovascular events; BMI, body mass index; eGFR, estimated glomerular filtration rate; CVD, cardiovascular disease; HR, hazard ratio; CI, confidence interval

Stratified analyses for the association between protein-adjusted serum free thiols and the risk of CV events showed consistently inverse associations in various subgroups, except for associations stratified by the presence of hypertension (*P*_interaction_ < 0.001) (Table 3; Fig. 4). Stratification by BMI and the presence of albuminuria, hypertension, and diabetes showed significant differences between groups. Corresponding HRs were lower for subjects with a lower BMI, without hypertension, and without diabetes. Conversely, HRs were lower in subjects with albuminuria.

**Table 3:** Stratified analyses for the association between protein-adjusted serum free thiols and the risk of cardiovascular (CV) events across various subgroups

Variable	CV events (*n*)	Total (*n*)	HR*	95% CI	*P*_interaction_
Overall	402	5955	0.68	0.47–1.00	**0.048**
Gender					
Female	87	2497	0.87	0.42–1.77	0.976
Male	221	2180	0.58	0.38–0.90	
BMI					
< 25.0	68	1910	0.53	0.23–1.21	**< 0.001**
> 25.0	239	2762	0.71	0.47–1.08	
Albuminuria					
No	237	4285	0.71	0.46–1.08	**0.008**
Yes	71	394	0.52	0.24–1.12	
Hypertension					
No	219	4143	0.55	0.36–0.86	**< 0.001**
Yes	94	501	1.06	0.51–2.19	
CVD history					
No	271	4526	0.69	0.46–1.04	0.327
Yes	37	155	0.47	0.18–1.21	
Diabetes					
No	289	4592	0.67	0.45–0.98	**0.012**
Yes	15	73	0.79	0.16–3.83	
Smoking					
No	200	3402	0.78	0.49–1.27	0.484
Yes	108	1279	0.46	0.26–0.83	
Alcohol consumption					
No	99	1134	0.72	0.38–1.36	0.911
Yes	209	3547	0.63	0.40–0.98	
Hypercholesterolemia					
No	260	4372	0.61	0.41–0.90	0.062
Yes	41	242	0.83	0.28–2.42	

Stratifications by BMI, albuminuria, hypertension, diabetes, and hypercholesterolemia showed significant differences between groups. Corresponding hazard ratios (HRs) are consistently lower for subjects with a more favorable cardiovascular risk profile (i.e., lower BMI and the absence of hypertension and diabetes), whereas the presence of CVD history or albuminuria shows lower HRs as well

*CV* cardiovascular, *HR* hazard ratio, *CI* confidence interval, *BMI* body mass index, *CVD* cardiovascular disease

*Adjusted for potential confounding factors (sex, age, hsCRP, current smoking, systolic blood pressure, total cholesterol, diabetes, and BMI)

**Fig. 5:**
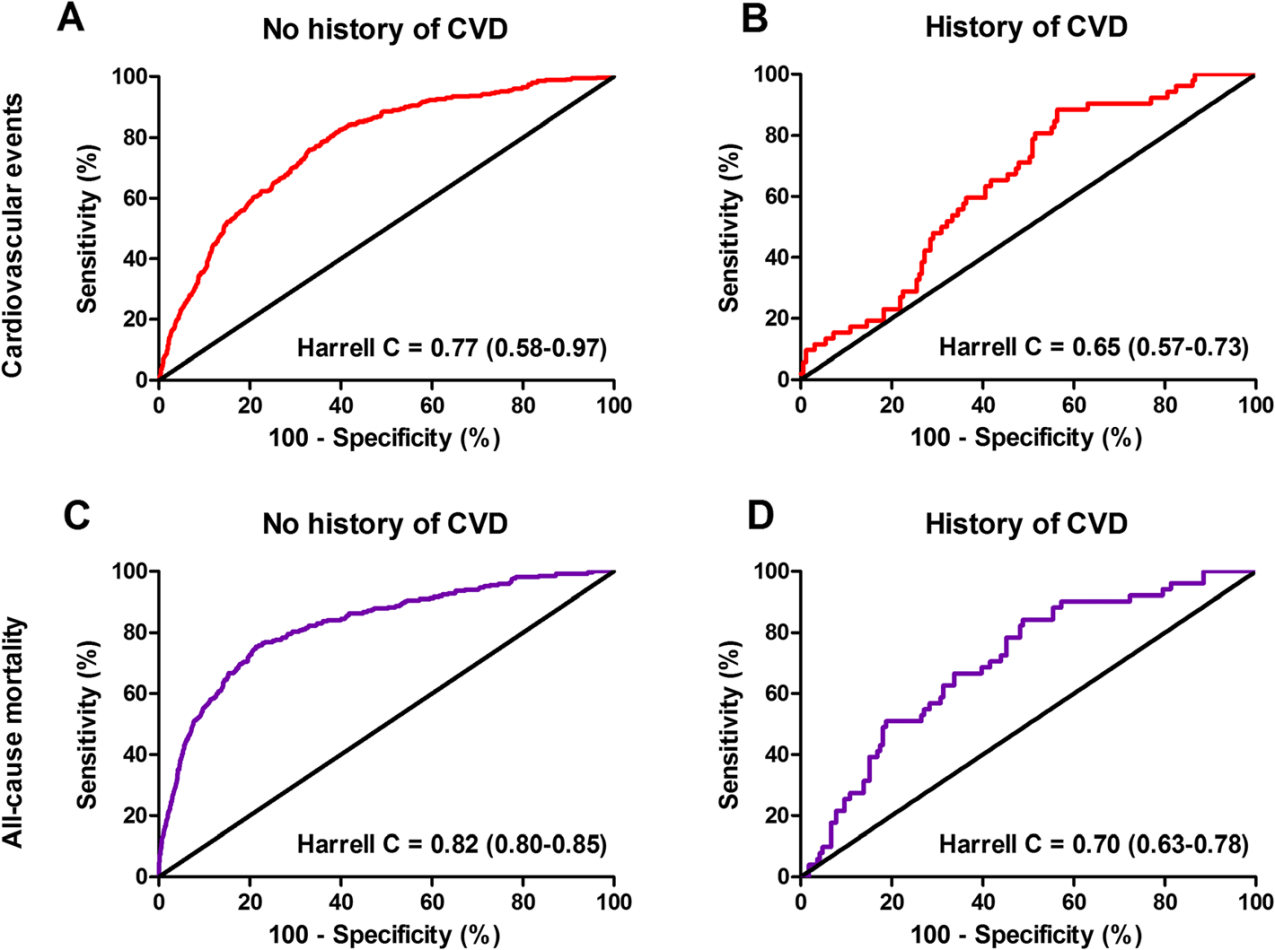
Receiver operating characteristic (ROC) curves with associated Harrell C indices (95% confidence interval [CI]) of Cox proportional hazards regression models in subjects with and without a history of cardiovascular disease (CVD). **a**, **b** Discriminative ability of Cox regression models for the association between protein-adjusted serum-free thiols and cardiovascular events in both subgroups. **c**, **d** Discriminative ability of Cox regression models for the association between protein-adjusted serum free thiols and all-cause mortality in both subgroups. CVD, cardiovascular disease

In subjects with a known history of CVD (*n* = 217, 3.6%), Cox proportional hazards regression analyses revealed no significant age- and sex-adjusted associations between protein-adjusted serum free thiol concentrations and the risk of CV events (Table 4 (A), HR per doubling of concentration 0.52 [0.23–1.19], *P* = 0.120). However, we observed significant age- and sex-adjusted associations in this subgroup between protein-adjusted serum free thiol concentrations and the risk of all-cause mortality when comparing the upper two tertiles with the lowest tertile (Table 4 (B), HR per doubling of concentration: tertile 2, 0.48 [0.24–0.96], *P* = 0.038; tertile 3, 0.40 [0.16–0.99], *P* = 0.047). Although there was a large difference in the sample size between subgroups of subjects with and without a history of CVD (*n* = 217, 3.6% vs. *n* = 5738, 96.4%, respectively), the discriminative capacity of these models (Harrell C indices) was higher in subjects without a known history of CVD compared to subjects with a history of CVD (Fig. 5).

**Table 4:** Cox proportional hazards regression models of the age- and sex-adjusted associations between protein-adjusted serum free thiols and the risk of (A) cardiovascular events and (B) all-cause mortality in participants with and without a history of cardiovascular disease (CVD)

	HR per doubling	Tertiles of protein-adjusted serum-free thiols		
		< 4.65 μmol/g	4.65–5.46 μmol/g	> 5.46 μmol/g
A. Cardiovascular events				
Total cohort	0.71 (0.51–0.97), ***P*** **= 0.031**	1.00 (reference)	0.94 (0.75–1.19), *P* = 0.600	0.82 (0.63–1.07), *P* = 0.135
No history of CVD	0.77 (0.54–1.08), *P* = 0.127	1.00 (reference)	0.96 (0.75–1.23), *P* = 0.761	0.83 (0.62–1.09), *P* = 0.181
History of CVD	0.52 (0.23–1.19), *P* = 0.120	1.00 (reference)	0.82 (0.42–1.61), *P* = 0.567	0.84 (0.38–1.86), *P* = 0.670
B. All-cause mortality				
Total cohort	0.71 (0.50–1.01), *P* = 0.056	1.00 (reference)	0.77 (0.59–1.00), ***P*** **= 0.048**	0.73 (0.54–1.00), ***P*** **= 0.049**
No history of CVD	0.80 (0.54–1.18), *P* = 0.253	1.00 (reference)	0.81 (0.61–1.09), *P* = 0.159	0.81 (0.58–1.12), *P* = 0.206
History of CVD	0.47 (0.22–1.02), *P* = 0.055	1.00 (reference)	0.48 (0.24–0.96), ***P*** **= 0.038**	0.40 (0.16–0.99), ***P*** **= 0.047**

All models were age- and sex-adjusted

*P* values in bold indicate statistical significance

*CVD* cardiovascular disease, *HR* hazard ratio

## Discussion

In this large population-based cohort, we found that protein-adjusted serum free thiol concentrations were able to significantly predict the risk of CV events and all-cause mortality. After adjustment for confounders, this predictive capacity of protein-adjusted serum free thiol concentrations (per doubling) regarding the risk of CV events, as well as the risk of all-cause mortality, remained statistically significant. Collectively, our results shed light on the potential significance of serum levels of free thiols for risk stratification in primary and secondary prevention settings to reduce CVD burden in the general population.

Over the last couple of decades, increasing attention has been paid to the role of oxidative stress in CVD, since it may lead to endothelial dysfunction [33]. For instance, it was previously shown that thiols are an oxidative stress marker in subjects with an acute myocardial infarction [34] and that plasma thiols are significantly lower in subjects with congestive heart failure, as compared to healthy subjects [35]. In the current study, we tested the hypothesis that protein-adjusted serum free thiols, due to the ROS-scavenging properties of free thiols, are markers of the systemic redox status that might be associated with the risk of CV events and mortality in the general population. Indeed, our results show that protein-adjusted serum free thiol concentrations (per doubling) are inversely associated with the risk of CV events after adjustment for potential confounders. Regarding mortality, after correcting for potential confounders, we observed a significant association with protein-adjusted serum free thiols. This postulation is in line with previous findings showing that serum thiols are independently associated with CV risk scores at the population level [36]. As compared to this cross-sectional study, however, the current study is much larger and of longitudinal origin (10 years of follow-up) and demonstrates associations of free thiols with the occurrence of CV events and all-cause mortality.

In our stratified analyses, the association between protein-adjusted serum free thiols and the risk of CV events showed no significant interaction between subjects with and without CVD. Moreover, we demonstrated that the association between protein-adjusted serum free thiols and the risk of CV events was consistently inversely associated in various subgroups, except for the association stratified by the presence of hypertension. Free thiols were found to be more negatively associated with CV events in subgroups characterized by the absence of obesity, hypertension, and diabetes. Conversely, free thiols were more negatively associated in subjects with albuminuria. Although we cannot clearly explain these findings, a possible explanation could be that albuminuria reflects ongoing endothelial damage that leads to a stronger prediction, whereas obesity and hypertension are merely risk factors and their presence does not significantly contribute to the predictive capacity. However, free thiols were more negatively associated with CV events in subjects without diabetes. Although it could be stated that subjects with diabetes might also show generalized manifest damage, the majority of these diabetic subjects had a relatively short disease duration. Therefore, we deem it unlikely that these subjects already had diabetes-induced microvascular complications. However, the value of free thiols in these specific subgroups requires further study as subgroup sizes were relatively unbalanced.

Free thiols (R-SH, sulfhydryl groups) are more accurately reflecting the systemic in vivo redox status as compared to many other individual oxidant or antioxidant factors and their derivatives [5]. In the extracellular environment, thiols are an integral part of a complex and dynamic redox signaling network and possess potent antioxidant activity based on their ability to rapidly scavenge circulating reactive species [18]. Extracellular thiols are predominantly embedded in plasma proteins, whereas a smaller percentage is represented by low-molecular-weight (LMW) thiols, such as cysteine, homocysteine, and glutathione (GSH) [37]. Blood proteins harbor the largest amount of redox-active thiol groups. In this respect, albumin is quantitatively the most important molecule within this pool (based on the existence of its single free cysteine residue, Cys^34^), given its abundance and because it is capable of transporting LMW thiols [18]. Collectively, systemic thiols circulate in both reduced and oxidized forms and, given their rapid and potent antioxidant activity, have a major impact on the net extracellular redox equilibrium. Therefore, measuring systemic free thiol availability may constitute a simple, robust, and easily reproducible method to assess the level of systemic oxidative stress in humans. Since proteins largely determine the total amount of measurable free thiol groups, adjusting these to circulating total protein or albumin levels can be regarded as an indirect approach to more precisely reflect the total serum free thiol pool [7, 8, 10, 11, 38].

Due to the fact that free thiols are known to be receptive to therapeutic modulation, our results shed light on the potential development of therapeutic interventions. For instance, several thiol-based antioxidants, such as *N*-acetylcysteine (NAC) or glutathione (GSH), have been investigated for their potential to restore extracellular thiol homeostasis in both human and animal studies [39]. Administration of these substances may increase the extracellular reducing capacity, though these potential effects should be carefully examined since they may disturb the diverse physiological functions of reactive species [40].

Strengths of the present study include the size and extensive clinical characterization of our study cohort and the long follow-up duration. However, several limitations also have to be taken into account. For instance, the observational nature of the study does not allow us to infer causality between serum levels of free thiols and CV events and all-cause mortality. In addition, given the fact that the majority of patients were Caucasians, the generalizability of our results in subjects with other ethnicities remains unknown. Likewise, the current study was carried out in the northern parts of the Netherlands; therefore, the generalizability of our results could be debated. Moreover, many arrows are absent in our directed acyclic graph (DAG) for which arguments for their presence could be made (for example, socio-economic status and BMI, socio-economic status and smoking, alcohol and BMI, smoking and BMI) [41–44]. However, adding these arrows as direct effects in Additional file 1: Figure S1 (scenario 3) would not have altered the set of confounders necessary to correct for in our analysis. In that sense, our analysis is robust to most of the underlying causal assumptions made.

## Conclusion

In conclusion, we show that protein-adjusted serum free thiol levels are significantly predictive of the risk of CV events and all-cause mortality in the general population. Although free thiols might harbor great potential as an easily measurable biomarker in the current primary and secondary CVD prevention strategies, future prospective studies will be required to further elucidate the clinical relevance of serum free thiol levels in this context.

## Supplementary information

**Additional file 1: Figure S1.** Directed Acyclic Graph (DAG) showing the causal paths which were hypothesized to be involved in the relationship between systemic oxidative stress and the risk of cardiovascular events and all-cause mortality. Arrows depict hypothesized causal (direct) effects between variables, whereas absence of an arrow between two variables represents the assumption of no such direct effect.

**Additional file 2: Figure S2 (A-B)**. Protein-adjusted serum free thiol concentrations (μmol/g) show no deviances from linear associations with either **(A)** the risk for CV events or **(B)** all-cause mortality. Estimated associations were derived from the adjusted Cox proportional hazards regression analysis (model 3) based on restricted cubic splines with three knots. *P*-values for non-linearity were *P* = 0.258 for CV-events and *P* = 0.642 for all-cause mortality. The median of protein-adjusted serum free thiol concentrations was taken as a reference standard (5.07 μmol/g of protein). Gray-shaded areas represent 95% confidence intervals.

## Data Availability

The datasets used and analyzed during the current study are available from the corresponding author on reasonable request.
