# Supplementary figures and images for: Serum free thiols predict cardiovascular events and all-cause mortality in the general population: a prospective cohort study
Source: BMC Med. 2020 May 27;18:130. doi: 10.1186/s12916-020-01587-w (PMC7251849; doi:10.1186/s12916-020-01587-w)

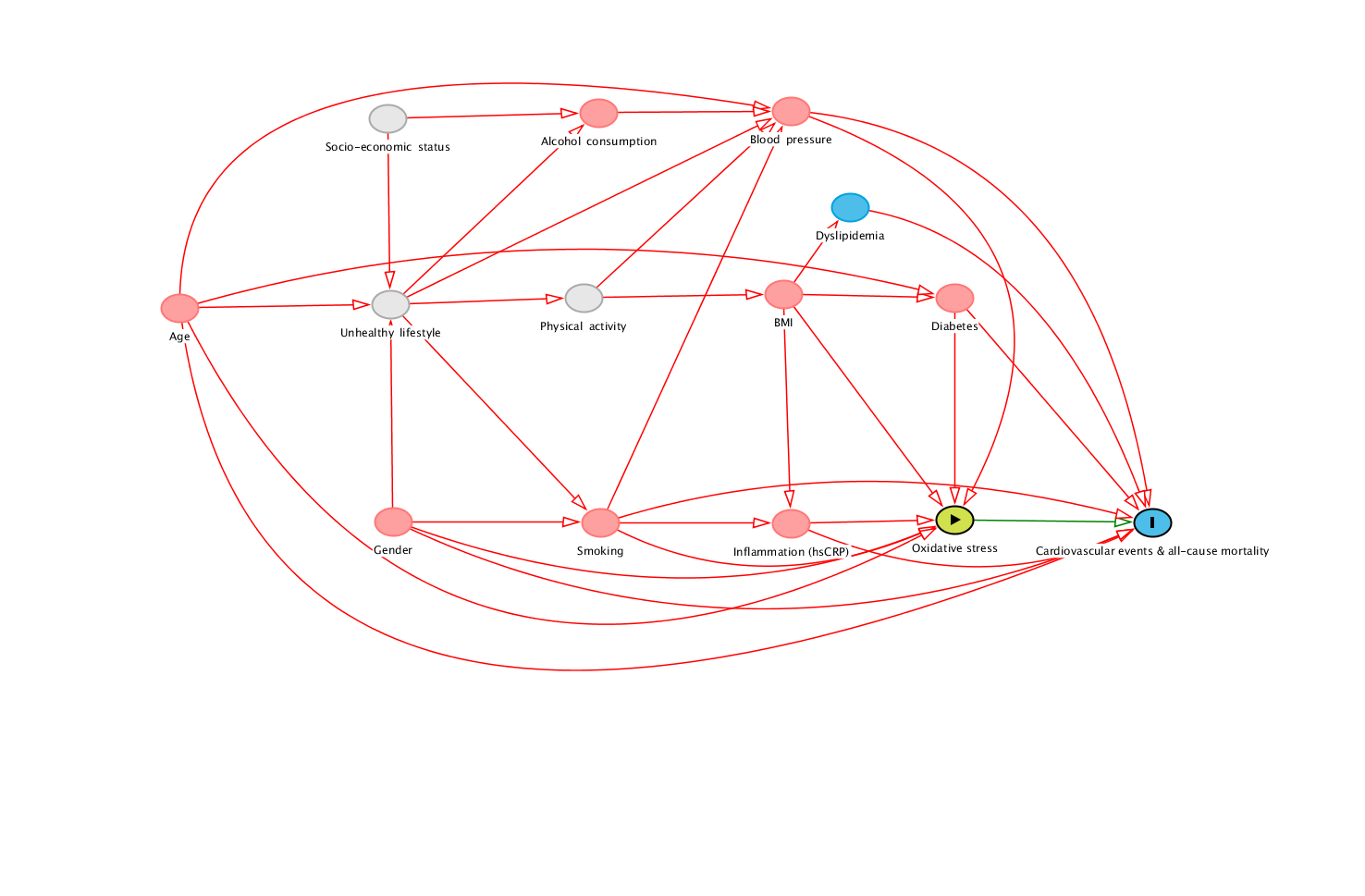

Supplement: Supplementary file 1 — Additional file 1: Figure S1. Directed Acyclic Graph (DAG) showing the causal paths which were hypothesized to be involved in the relationship between systemic oxidative stress and the risk of cardiovascular events and all-cause mortality. Arrows depict hypothesized causal (direct) effects between variables, whereas absence of an arrow between two variables represents the assumption of no such direct effect. [file 12916_2020_1587_MOESM1_ESM.png]

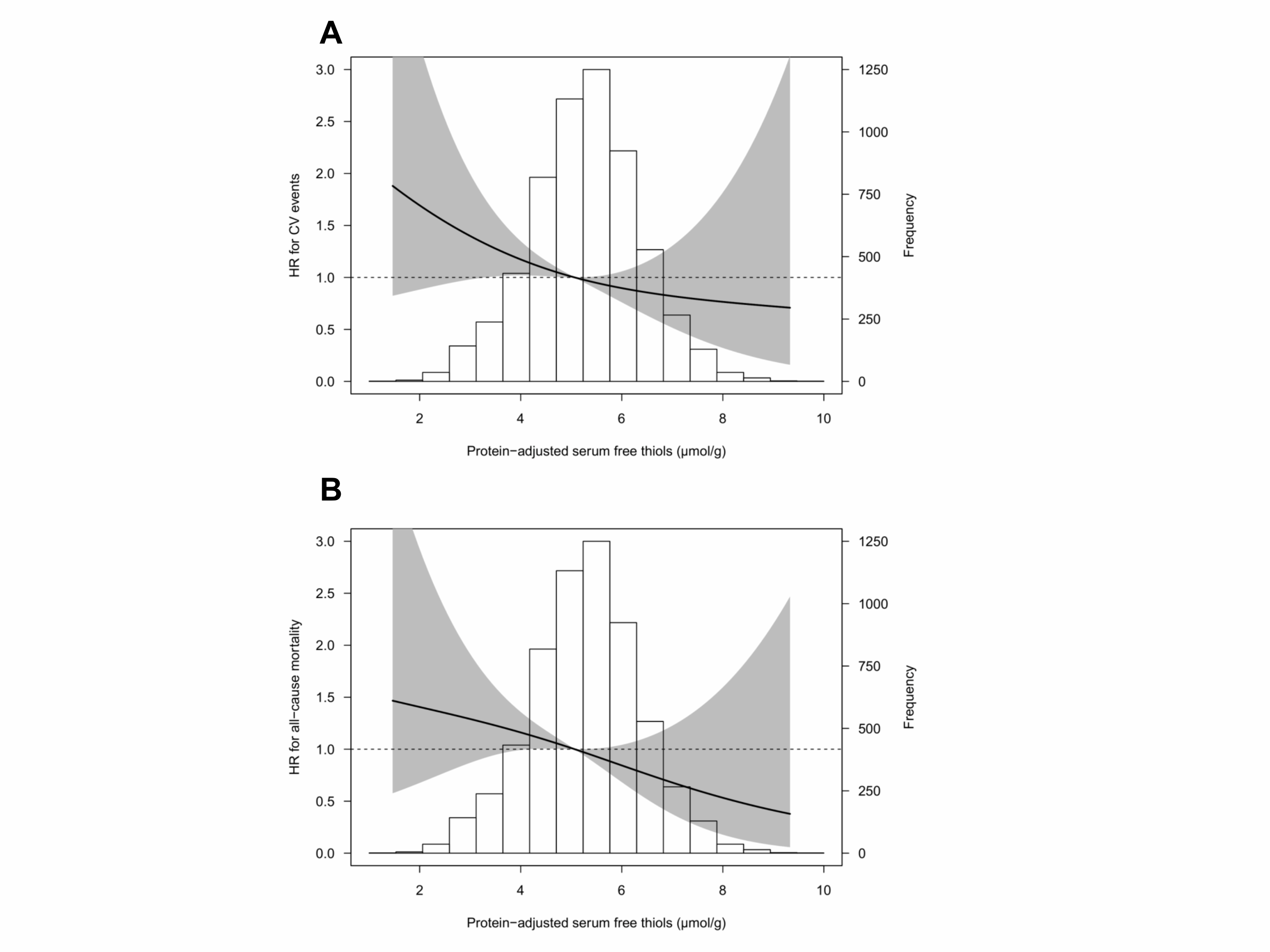

Supplement: Supplementary file 2 — Additional file 2: Figure S2 (A-B). Protein-adjusted serum free thiol concentrations (μmol/g) show no deviances from linear associations with either (A) the risk for CV events or (B) all-cause mortality. Estimated associations were derived from the adjusted Cox proportional hazards regression analysis (model 3) based on restricted cubic splines with three knots. P-values for non-linearity were P = 0.258 for CV-events and P = 0.642 for all-cause mortality. The median of protein-adjusted serum free thiol concentrations was taken as a reference standard (5.07 μmol/g of protein). Gray-shaded areas represent 95% confidence intervals. [file 12916_2020_1587_MOESM2_ESM.tiff]
